# Insights into the differences related to the resistance mechanisms to the highly toxic fruit *Hippomane mancinella* (Malpighiales: Euphorbiaceae) between the larvae of the sister species *Anastrepha acris* and *Anastrepha ludens* (Diptera: Tephritidae) through comparative transcriptomics

**DOI:** 10.3389/fphys.2024.1263475

**Published:** 2024-01-18

**Authors:** Essicka A. García-Saldaña, Daniel Cerqueda-García, Enrique Ibarra-Laclette, Martín Aluja

**Affiliations:** ^1^ Clúster Científico y Tecnológico BioMimic^®^ , Red de Manejo Biorracional de Plagas y Vectores, Instituto de Ecología, A C–INECOL, Xalapa, Veracruz, Mexico; ^2^ Clúster Científico y Tecnológico BioMimic^®^ , Red de Estudios Moleculares Avanzados, Instituto de Ecología, A C–INECOL, Xalapa, Veracruz, Mexico

**Keywords:** Herbivory, detoxification mechanisms, transcriptomics, *Anastrepha acris*, *Anastrepha ludens*, Diptera: Tephritidae, *Hippomane mancinella*

## Abstract

The Manchineel, *Hippomane mancinella* (“Death Apple Tree”) is one of the most toxic fruits worldwide and nevertheless is the host plant of the monophagous fruit fly species *Anastrepha acris* (Diptera: Tephritidae). Here we aimed at elucidating the detoxification mechanisms in larvae of *A. acris* reared on a diet enriched with the toxic fruit (6% lyophilizate) through comparative transcriptomics. We compared the performance of *A. acris* larvae with that of the sister *species A. ludens,* a highly polyphagous pest species that is unable to infest *H. mancinella* in nature. The transcriptional alterations in *A. ludens* were significantly greater than in *A. acris*. We mainly found two resistance mechanisms in both species: structural, activating cuticle protein biosynthesis (chitin-binding proteins likely reducing permeability to toxic compounds in the intestine), and metabolic, triggering biosynthesis of serine proteases and xenobiotic metabolism activation by glutathione-S-transferases and cytochrome P450 oxidoreductase. Some cuticle proteins and serine proteases were not orthologous between both species, suggesting that in *A. acris*, a structural resistance mechanism has been selected allowing specialization to the highly toxic host plant. Our results represent a nice example of how two phylogenetically close species diverged over recent evolutionary time related to resistance mechanisms to plant secondary metabolites.

## 1 Introduction

Insects are the most diverse group of animals on earth with over a million documented species, many being essential for ecosystem functionality (Grimaldi and Engel, 2005). Their ecological significance spans from nutrient cycling and plant pollination to serving as food sources for many taxa (Scudder, 2017). Moreover, certain insect species have profound economic impacts given their pest status and the interactions that ensue when completing their life cycles in commercially valuable plants/fruits (Lemelin et al., 2016). One such interaction is the resistance/tolerance to the toxic compounds found in certain fruits, which partially protect plants from herbivores (Weng et al., 2012). Understanding the resistance mechanisms to these toxic compounds can provide insights into insects' evolutionary adaptations and survival strategies, especially when considering closely related species (Aluja and Mangan, 2008; Ali and Agrawal, 2012; Giron et al., 2018).

A particularly important group of insects is represented by the true fruit flies (Diptera: Tephritidae) which includes more than 5,000 species (Norrbom, 2010; Liquido et al., 2019). As part of their life history, fruit fly females lay their eggs inside their host’s pulp or seeds, where they find favorable conditions for the growth and development of their larvae (Aluja and Mangan, 2008). This behavior causes certain fruit fly species to affect a wide range of commercially valuable fruit crops, limiting international trade of agricultural products (Aluja and Mangan, 2008). In fact, some of these fruit flies are listed among the fruit-tree pest insect species with the greatest economic impact worldwide (White and Elson-Harris, 1992). Among these species, those belonging to the *Anastrepha* genus are one of the most relevant in Mexico and the Neotropics (Hernández-Ortiz et al., 2010). Such is the case of *Anastrepha ludens* (Loew) (Diptera: Tephritidae), commonly known as the Mexican Fruit Fly.

*Anastrepha ludens* is classified as a highly polyphagous pest, due to its feeding habits which confer it the ability to attack many plant species of several families, including many economically important fruit species such as mango (*Mangifera indica* L.; Anacardiaceae), citrus (*Citrus aurantium* L. or *Citrus x sinensis* L.; Rutaceae), peach (*Prunus persica* L.; Rosaceae), and pepper (*Capsicum pubescens* Ruiz y Pav.; Solanaceae) (Birke et al., 2015; Birke and Aluja, 2018); or wild fruit species, including *Casimiroa edulis* La Llave and Lex (also known as white sapote or matasano) and *C. greggii* (S. Watson) F. Chiang (commonly known as yellow chapote), both within the Rutaceae family. These last two species are the purported ancestral hosts of *A. ludens* (Birke et al., 2015).

In contrast to highly polyphagous tephritid species (*i.e.*, *A. ludens*, the Oriental fruit fly - *Bactrocera dorsalis* Hendel, or the Mediterranean fruit fly - *Ceratitis capitata* Wiedemann), there are other fruit fly species with strict monophagous feeding habits. A good example of a monophagous fruit fly is represented by *B. oleae* Gmelin, commonly known as the Olive Fly. This fly specializes in infesting the fruit of *Olea europaeae* L (olive tree), which contains high levels of phenolic compounds (up to 14% dry weight-based), many of them being active against insects (Ben-Yosef et al., 2015). Among these compounds, Oleuropein, a glucid that is lethal to *Bactrocera oleae* larvae, stands out. Despite this, the larvae of *B. oleae* can develop optimally in the fruit of the olive tree, because it hosts the bacterium *Candidatus Erwinia dacicola* (Enterobacteriaceae), a symbiont found in its digestive tract that assists the larvae to catabolize oleuropein (Ben-Yosef et al., 2015). A similar association between a key bacterium degrading toxic polyphenols (*i.e.*, *Komagataeibacter*) and a fruit fly (the stenophagous *A*. *striata*) was recently reported (Ochoa-Sánchez et al., 2022). Here we chose as study model another fruit fly species adapted to monophagy: *Anastrepha acris* Stone (Diptera: Tephritidae), which is phylogenetically very close to *A. ludens* (Mengual et al., 2017), but exclusively infests fruits of *Hippomane mancinella* L (Malpighiales: Euphorbiaceae) (Aluja and Norrbom, 2000; Aluja et al., 2020). *Hippomane mancinella* is a tree that produces a fruit commonly known as “Apple of Death", which is highly toxic to many animals, including humans and insects (Rao, 1974; Adolf and Hecker, 1984).

Recently, Aluja et al. (2020) analyzed the interaction between *H. mancinella, A. acris,* and *A. ludens*, and found that diets enriched with different concentrations of dried fruit pulp of *H. mancinella* have a differential effect on the development of the larvae of both species. Not surprisingly, in *A. ludens* they found that *H. mancinella* produces a more pronounced, negative impact on larvae development as there is no natural association between the fly and this toxic fruit in nature. For example, only 0.08% of *A. ludens* larvae developed into pupae in a diet supplemented with 12.5% *H. mancinella* compared with *A. acris* larvae, which fully metamorphosed into pupae and viable adults. However, the number and weight of the exposed pupae decreased as concentration increased. This study nicely complemented the classical literature on the topic of specialized insects dealing with toxic plants (*e.g.*, Berenbaum et al., 1989; Adler et al., 1995; Zalucki et al., 2001; Agrawal and Kurashige, 2003; Harvey et al., 2007). It becomes clear that monophagous species (*i.e.*, highly specialized ones) are not totally immune to plant defense, but they have developed physiological adaptations which allow individuals to cope with deleterious allelochemicals contained in their hosts, exhibiting greater tolerance to these chemicals than polyphagous species. The active compounds reported in *H. mancinella* include the ellagitannins Hippomanin A and B, phenylpropanoids, coumarins, and flavonoids (*e.g.*, naringenin, kaempferol, hesperidin, and quercetin) (Rao, 1974; Aluja et al., 2020). Hippomanin A (a hydrolyzable tannin) is the main active, toxic compound of *H. mancinella* fruit. When it undergoes acid hydrolysis, it yields glucose, ellagic acid, and gallic acid (Rao, 1974; Rao, 1977). However, Hippomanin A was not detected in any of the developmental stages of *A. acris* and neither in its parasitoid; *Doryctobracon areolatus* (Szépligeti) (Hymenoptera: *Braconidae*), which suggests that *A. acris* developed detoxification mechanisms to metabolize it (Aluja et al., 2020).

Despite their economic importance, there is still scant work aimed at understanding the mechanisms used by fruit flies to defend themselves from the toxic specialized metabolites of their plant hosts. In this sense, gene expression studies can be helpful in our quest to elucidate the metabolic impact of plant compounds on herbivorous insects and their effects on their feeding behavior and development (Thitz et al., 2020). For example, Wang et al. (2020) and Zhao et al. (2022) tested the reaction of *Spodoptera litura* (Fabricius) (Lepidoptera: Noctuidae) fed on a diet enriched with tannins. They found that the genes encoding for proteins involved in xenobiotic metabolism (*e.g.*, cytochrome, and glutathione-related proteins) and digestive enzymes (*e.g.,* lipases and carbohydrases), were highly regulated in the midgut upon diet intake. In *Helicoverpa armigera* (Hübner) (Lepidoptera: Noctuidae) moth, Zheng et al. (2022) evaluated the transcriptomic changes in response to gossypol and tannic acid and identified several transcript-encoding-enzymes involved in immunity, digestion, and detoxification metabolism, including glutathione S-transferases (GSTs), UDP-glucosyl-transferases (UGTs), hydrolases, serine-proteases, lipases, Hsp20, or aldehyde dehydrogenases, explaining how tannins are degraded within the digestive tract of the insect (Zheng et al., 2022). Celorio-Mancera et al. (2011) evaluated the defensive mechanisms of the generalist insect *H. armigera* against the toxic compounds produced by plants within the genus *Gossypium* L (Malvales: Malvaceae). These authors determined that some phytoregulators (*i.e.*, jasmonic acid, salicylic acid, and ethylene) and phenolic compounds modified the transcriptional responses of the larvae at the primary metabolism level and activated its stress response (Celorio-Mancera et al., 2011).

Based on all the above, but particularly following the emphatic call by Ali and Agrawal (2012) recommending the study of related insects with contrasting feeding habits to foster a deeper understanding of the many mechanisms behind herbivory, here we applied a comparative transcriptomic approach to identify the response of *A. acris* and *A. ludens* larvae when fed a lyophilized extract of *H*. *mancinella.* The latter, to gain insight into the resistance and detoxification mechanisms used by these sister species to overcome the effects of toxic phytochemicals. Based on the “phytochemical coevolution theory/hypothesis” (Cornell and Hawkins, 2003), we predicted that albeit their close phylogenetic relationship, *A*. *acris*, the strict specialist, would have evolved resistance/degrading mechanisms that the congener, the broad generalist, either lost or never developed during evolution.

## 2 Materials and methods

### 2.1 Biological material

To obtain wild specimens of *A. acris* and *A. ludens*, we made collections of infested host fruit in nature. For this, we collected a total of 65 kg of *H. mancinella* (host of *A. acris*) and 60 kg of *Citrus x aurantium* (bitter orange; host of *A. ludens*). The first were collected at Punta Mita (20° 46′19.58“N, 105° 30′38.79″W) and Lo de Marcos (20° 57′13.28“N, 105° 21′59.09″W) in the state of Nayarit, Mexico during July 2020. Infested *C. x aurantium* fruits were collected in September 2020, in Alborada, Veracruz, Mexico (19° 26′49.8“N, 96° 52′24.5″W). The criteria used to determine infestation status in fruit was the identification of small holes in the fruit skin or brown spots (indicating fruit decomposition), both of which are signs of oviposition and larval feeding activity inside the fruit. In addition, 15 kg of uninfested *H. mancinella* fruit were also collected and frozen in liquid nitrogen in the field. These samples were used to lyophilize the pulp used for the experimental diets, following the protocol described by Aluja et al. (2020).

The field-collected material was processed in the laboratories of the Biorational Pest and Vector Management Network (RMBPV), embedded in the BioMimic^®^ Scientific and Technological Cluster, Instituto de Ecología, A.C. (INECOL) in Coatepec, Veracruz, Mexico. Methods described in Aluja et al. (2020) were followed to process infested fruit and collect the larvae/pupae yielded by the fruit.

### 2.2 Egg collection

Adult and sexually mature flies of each species were distributed in 15 30 × 30 × 30 cm transparent acrylic cages, at a density of 30 ♀ and 15 ♂´s (a total of 450 ♀ and 225 ♂). Inside each cage, we placed containers maintained with water, food (*i*.*e*., hydrolyzed protein and sugar in a 1: 3 w/w ratio), with a photoperiod of 12:12 h L:D, at 27°C ± 1°C and 70% ± 5% of relative humidity (RH). For the egg collection, a green cloth, balloon-shaped oviposition device was introduced into each cage, filled with a 0.2% sodium benzoate solution as conservative (Pascacio-Villafán et al., 2018). The devices placed in the *A. acris* cages were impregnated with a mixture of sterile water and 50% of the volume with lyophilized pulp of *H. mancinella* because preliminary observations showed that females do not oviposit in *H. mancinella*-free mixtures. Devices used for *A. ludens* oviposition were free of the *H. mancinella* lyophilizate. Oviposition devices (*i.e.,* egg-collection devices) were retrieved every day after exposing them to flies for 24 h, until the flies stopped producing offspring/eggs (*i.e.*, approximately two and a half months for both species). The eggs were placed on the top of a black cloth placed over moistened cotton, with 5 mL of 0.2% sodium benzoate in plastic Petri dishes (6 cm in diameter) and were incubated in the dark at 29°C ± 1°C and 70% ± 5% RH. The eggs hatched after ca. Three days and the larvae were transferred to Petri dishes (6 cm in diameter) containing the experimental diets described below.

### 2.3 Transcriptomic experiment

To compare the gene expression of *A. acris* and *A. ludens* larvae in response to lyophilized pulp of *H. mancinella*, we mixed 6% of the lyophilizate with an artificial diet. The artificial diet consisted of a mixture of guar gum (0.096%), nipagin (methylparaben, 0.096%), sodium benzoate (0.38%), citric acid (0.42%), corn flour (5.1%), yeast (5.78%), sugar (7.9%), cob powder (18.3%) and distilled water (61.88%) (as described in Aluja et al., 2020). We determined the concentration used in this study based on a previous one (Aluja et al., 2020), where we reported that *A. acris* larvae can survive until the pupal stage with negligible negative effect up to a concentration of ∼30% of *H*. *mancinella* pulp mixed with an artificial diet, but *A. ludens* larvae reached their survival limit when exposed to a proportion of 12.5% of pulp in the artificial diet (>99% of the larvae died). Therefore, here we used the sub-lethal dose of 6% of lyophilized pulp as the experimental treatment to be able to compare both species.

In the case of the control treatment, larvae were fed with the same artificial diet but without the addition of the toxic *H. mancinella* lyophilizate. This control setup was crucial in establishing baseline gene expression levels in both species under standard dietary conditions. Fifty newly eclosed larvae of each fly species were placed in a 60 mm diameter Petri dish, with 16 g of diet (artificial diet with 6% or without *H. mancinella* lyophilizate). These larvae were kept at 29°C ± 1°C, 70% ± 5% of RH, and were fed for 10 days, until the third instar. Three larvae were taken from each Petri dish and preserved in five volumes of RNAlater^®^ (Qiagen), then they were frozen in liquid nitrogen and kept at −80°C (Oppert et al., 2012).

Formal transcriptomic analyses considered three larvae × three replicates × two conditions = 18 larvae per species (36 larvae in total). Each replicate consisted of a pool of three larvae. Replicates were collected during identical but independent and randomized experimental runs, with independent observations, but with strictly controlled environmental conditions. RNA extractions were made with the RNeasy^®^ Plant mini-Kit (Qiagen^©^; Hilden, Germany). Total RNA concentration was quantified in a NanoDrop^®^ spectrophotometer (ND-1000, NanoDrop Technologies; Wilmington, DE, United States), while its integrity was verified on 1% agarose gel.

Prior to sequencing, the RIN (RNA integrity number) of each sample was estimated using the Agilent 2,100 Bioanalyzer^®^ capillary electrophoresis system (Agilent Technologies^©^; Palo Alto, CA, United States). Only samples with a RIN >8 were considered for sequencing. The construction of the libraries was achieved with the TruSeq^®^ RNA Sample Preparation v2 kit (Illumina^®^; San Diego, CA). Each library was normalized to a final concentration of 20 mM and was sequenced on NextSeq500^®^ equipment (Illumina^©^; San Diego, CA), using a paired 2 × 150 bp format, at the sequencing unit of the Instituto de Ecología, A.C. (INECOL).

### 2.4 Bioinformatic analysis

The analysis workflow to process the Illumina paired-end (2 × 150) raw reads was performed in the High-Performance Cluster at the Instituto de Ecología A.C. (INECOL; Xalapa Veracruz, Mexico). A *de novo* transcriptome assembly was performed for each species. For each library, the Illumina adapters were removed, the first 10 bp of the raw reads were trimmed, and the low-quality sequences were cleaned, considering a minimum QScore quality value of 20, using the Trim galore v0.4.5 (Krueger, 2012) and Cutadapt v1.9.1 (Martin, 2011) software. The sequencing quality of each dataset was checked using the FastQC program (http://www.bioinformatics.babraham.ac.uk/projects/fastqc). A single *de novo* transcriptome assembly was generated for each species using the Trinity software with default parameters (Grabherr et al., 2011). After the assembly, we obtained the unigenes or transcripts which were processed with the SeqClean program to remove the poly A tails (https://sourceforge.net/projects/seqclean). To reduce redundant sequences and errors generated by insertions or deletions in the transcriptomes of each species, the coding regions of the unigenes were identified and the reading frames were corrected with the AlignWise software, taking as reference an insect’s genomes database (Evans and Loose, 2015). To reduce redundancy, unigenes were clustered at 95% similarity with the CD-HIT program (Li and Godzik, 2006). To estimate the transcripts’ abundance, the raw libraries were mapped with the Salmon software against the transcriptome assembly of their respective species (Patro et al., 2017). The differentially expressed unigenes (DEGs) were calculated with DEseq2 software (Love et al., 2014) in the R Studio 4.1.0 under R (R Development Core Team, 2021). We focused on contrasting the gene expression between control and treatment groups to identify Differentially Expressed Genes (DEGs). Initially, we filtered out transcripts exhibiting less than 10 transcripts per million (tpm). Subsequently, for the identification of DEGs, we applied a threshold criterion were unigenes exhibiting a fold change (FC) of ≥1 or ≤ −1, coupled with an adjusted *p*-value (*p*-adj, corrected for False Discovery Rate, FDR) of less than 0.05, were considered significant. This approach allowed us to robustly determine genes that were significantly upregulated or downregulated in the treatment group compared to the control. To explore the results, the up and downregulated set of DEGs per experiment were visualized as volcano plots, Pearson´s correlations, PCAs, heatmaps, and pie charts, with the R Studio 4.1.0 software (R Development Core Team, 2021), using the ggrepel (Slowikowski, 2021), EnhancedVolcano (Blighe et al., 2021), magrittr (Milton-Bache and Wickham, 2020), corrplot (Wei and Simko, 2021), pcaExplorer (Marini and Binder, 2019), cluster (Maechler et al., 2021), pheatmap (Kolde, 2019), dplyr (Wickham et al., 2021), ggplot2 (Wickham, 2016) and ggpubr (Kassambara, 2020) packages. Then, the protein IDs and the amino acid sequences were obtained from the sets of DEGs, to obtain the functional annotation. The functional annotation assignments were performed using the eggNOG-mapper website (Cantalapiedra et al., 2021), specifying a minimum hit e-value = 0, minimum hit bit-score = 60, Percentage identity = 40, minimum % of query coverage = 40, minimum % of subject coverage = 40, only transferring annotations from one-to-one orthology, with experimental evidence only and realigning queries to the whole PFAM DB. The Pathway-tool 13.0 software (Karp et al., 2010) was used to perform the metabolic reconstruction. For this, we used the gff3 annotation file obtained from eggNOG. The reconstruction was done with the PathoLogic algorithm, using the taxonomic pruning option to avoid false positives or pathways not present in tephritids, with a pathway prediction score cutoff of 0.15. Those reconstructed pathways associated with differential genes were identified and their log_2_ (FC) was plotted as a heatmap.

We used the heuristic tool Proteinortho (Lechner et al., 2011) to detect orthology between coding proteins of *A. ludens* and *A. acris*. Input data were the aminoacid sequences codified by the corrected ORFs by AlignWise (Evans and Loose, 2015). We used “Proteinortho parameters” as default (Lechner et al., 2011).

## 3 Results

### 3.1 *De novo* transcriptome assembly

The minimum and maximum number of raw reads obtained in the libraries of *A. acris* was 21,886,356 and 27,284,901 (with a mean of 24,776,710.3 and a standard deviation 1,983,057.6), and for *A. ludens* was 22,962,425 and 26,517,707 (with a mean of 24,936,771.2 and standard deviation 1,455,172.4). After trimming the mean number of reads was 24,727,965.8 (standard deviation 1,984,655.3) for *A. acris* and 24,896,660.5 (standard deviation 1,451,546.2) for *A. ludens*. The statistics of the transcriptomes of both species are shown in Table 1.

**TABLE 1:** General statistics of the *de-novo* transcriptome assembly of *A. acris* and *A. ludens*.

Parameters	*A. acris*	*A. ludens*
Total of unigenes (“genes”)	112,572	141,660
Total of transcripts (“contigs or transcripts”)	187,958	232,187
Percent GC	39.21%	39.12%
Transcripts/contigs with N50 (bp)	1834	1,491
Median contig length (bp)	403	379
Median unigene length (bp)	344	322
Mean contig length (bp)	886.53	787.92
Mean unigene length (bp)	653.02	591.52
Total assembled bases of contigs	166,629,799	182,945,902
Total assembled bases of unigenes	73,511,905	83,794,024
Unigenes >10 tpm	29,592	32,216
DEGs downregulated	31	64
DEGs upregulated	22	184
Percentage of DEGs downregulated with annotation	87.1%	93.7%
Percentage of DEGs upregulated with annotation	97.4%	80.4%
DEGs downregulated with COG	25	60
DEGs upregulated with COG	21	147
DEGs downregulated with Pfam	23	58
DEGs upregulated with Pfam	10	146

### 3.2 Transcriptome overview of DGEs found in *A. acris* and *A. ludens*

**FIGURE 1:**
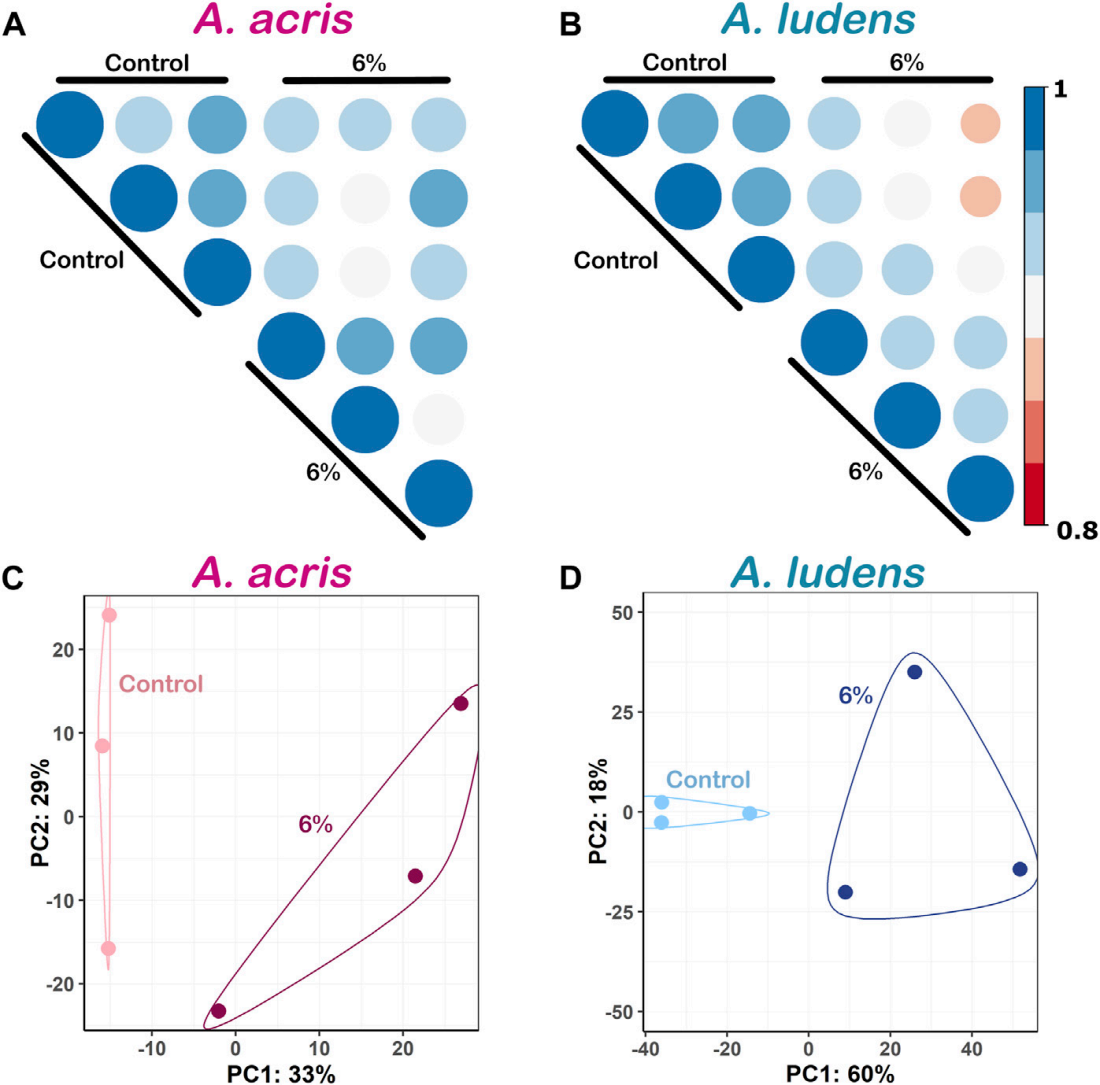
Exploratory plots of the transcriptomic differences between the species *A. acris* and *A. ludens* and between treatments. **(A)** and **(B)** Pearson’s correlation analysis of the DEGs between the replicas. All replicas had correlation values >0.8. **(C)** and **(D)** Principal component analysis (PCA) of the DEGs from both species. Each point represents a biological replica. The values in the *y* and *x*-axes represent the variation percentages explained by their respective main components.

In the PCA analysis, libraries generated from both *A. acris* and *A. ludens* were clustered according to the treatments (highlighted in circles). Respectively, 33% and 60% of the observed variance were explained by the first of two components. While the separation between the variables was evident (Figures 1A, B), no replicates were detected as outliers (Figures 1C, D).

**FIGURE 2:**
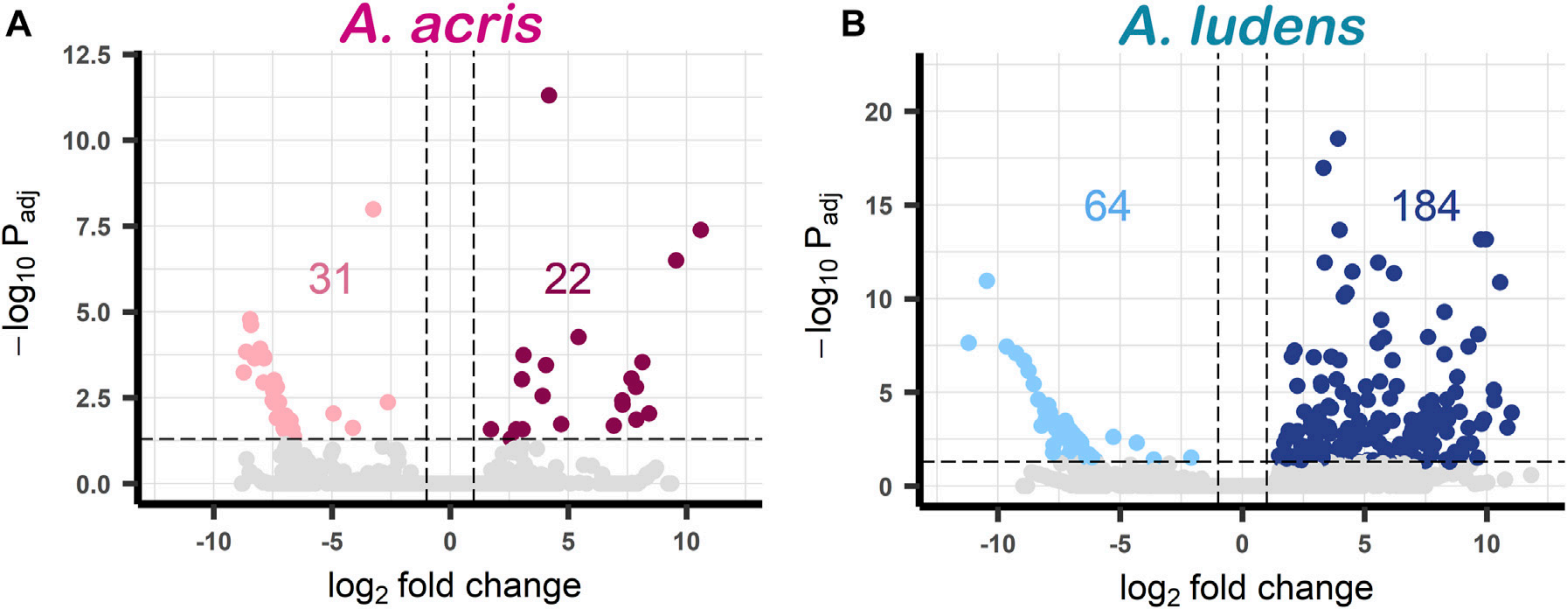
Volcano plots of DEGs found in **(A)** *A. acris* and **(B)** *A. ludens* in response to 6% *H. mancinella* lyophilizates mixed in an artificial rearing medium. The dotted horizontal line indicates the–log Padj = 0.05 and the dotted vertical lines indicate the log₂ Fold change ≥1 and ≤ −1. The statistically significant DEGs are colored points (reds and blues). On the left side those that were repressed “downregulated” (pink and light blue) and on the right the overexpressed unigenes “upregulated” (cherry and navy blue) are highlighted. Non-DEGs are shown in gray.

**FIGURE 3:**
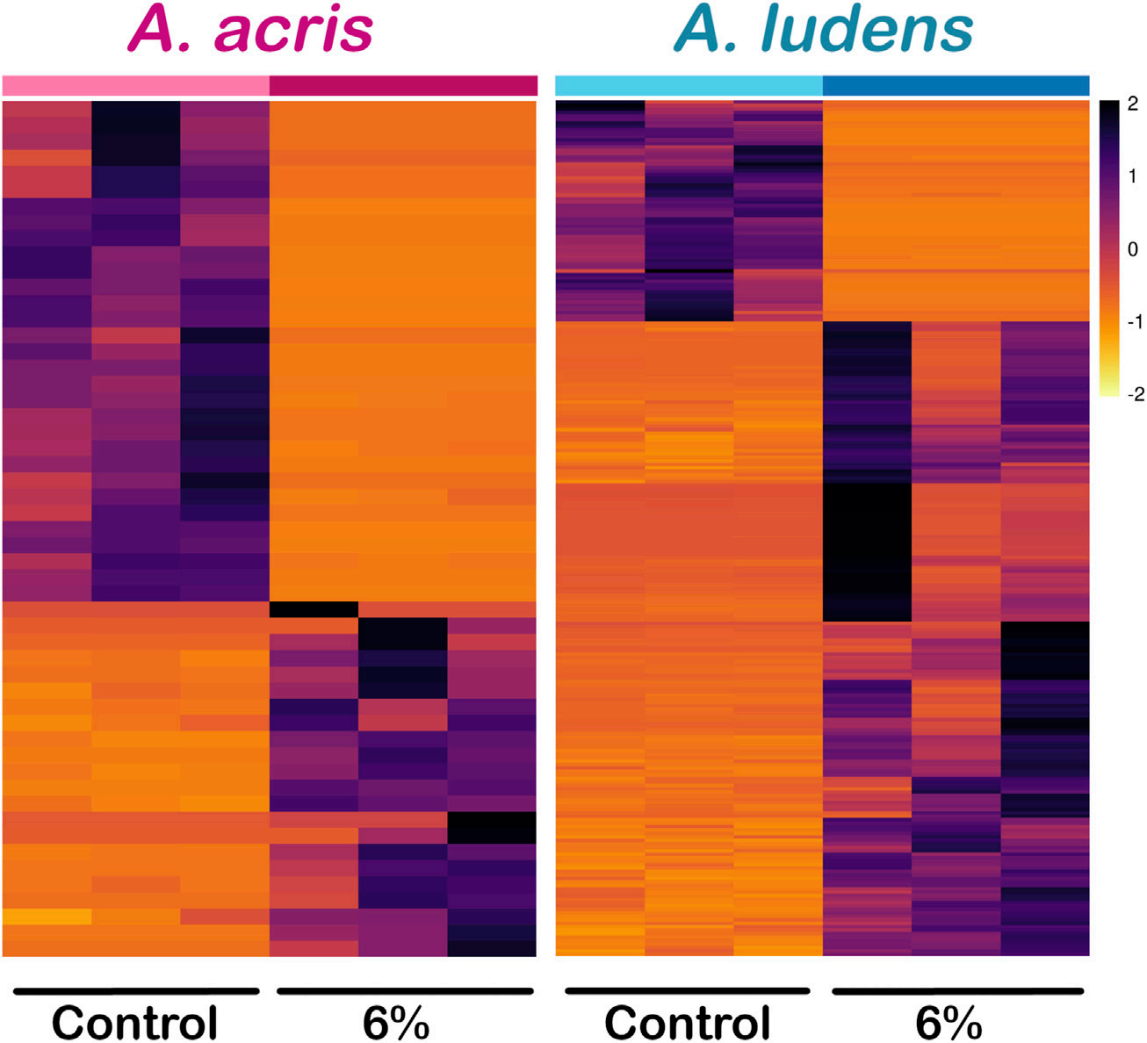
Heatmap of differentially expressed unigenes (DEG) of *A. acris* and *A. ludens* exposed to 6% *H. mancinella* lyophilizates mixed in an artificial rearing medium. The scale indicates the log₂. Fold change of each of the DEGs and each column corresponds to a replica.

Comparing the transcripts expressed by the larvae developed in the diet enriched with *H. mancinella* lyophilizates, we found a greater number of deregulated unigenes in *A. ludens*, compared to *A. acris*: 64 unigenes were repressed (downregulated) and 184 were overexpressed (upregulated) (with a log₂ Fold change ≥ and ≤1) (Figures 2A, B). Much fewer transcripts were altered in *A. acris*: 31 were repressed and 22 overexpressed (Figures 2A, B). We observed a clear separation between up and downregulated unigenes in the heatmaps in both species that developed in a *H. mancinella* enriched diet. Importantly, the three replicates of each species indicate reproducibility (Figure 3).

In both species, DEGs were related mainly to cuticle biosynthesis, hypoxia, and xenobiotic metabolism. However, *A. ludens* exhibited a higher number of DEGs compared to *A. acris* (Table 2). In both species, several transcripts encoding for chitin-binding and cuticle structural constituent proteins (Chitin_bind_4, Cuticle_4) were identified (in *A. acris* a total of 4 and 40 in *A. ludens*), as well as transcripts encoding hemocyanin proteins (Hemocyanin_C, Hemocyanin_M, Hemocyanin_N) (in *A. acris* a total of 2 and 10 in *A. ludens*). We detected several upregulated transcripts involved in the metabolism of xenobiotics (mainly in Phase one of detoxification) and with redox activity such as P450, COX3, Cytochrom_B, GST_C, GST_N, 3HCDH, adh_short_C2, Aldo_ket_red and Malic_M (in *A. acris* a total of 3 and 19 in *A. ludens*). Also, we detected the upregulation of transcripts encoding for Trypsin and serine proteases involved in digestive processes as well as in the proteolytic activation of zymogens, such as Prophenoloxidase (in *A. acris* a total of 3 and 21 in *A. ludens*) (Table 2). However, only in *A. ludens* did we detect the upregulation of transcripts encoding proteins related to fermentative pathways, such as G6PD, 6PGD-NAD_binding. In *A. ludens*, the downregulated transcripts are related to signal transduction processes, transport, ubiquitination, and proteins with kinase activity, such as PI3K, FAT, DUF, and HECT. In *A. acris*, repressed transcripts include those encoding for SNARE proteins, involved in the fusion of vesicles with the membrane (Table 2). Please note that we are providing a Supplementary Data Sheet that includes the differentially expressed genes for both species with their log fold changes, annotations, and nucleotide sequences.

**TABLE 2:** Annotation of the relevant up and downregulated DEGs identified in *A. acris* and *A. ludens* larvae in response to 6% *H. mancinella* lyophilizates mixed in an artificial rearing medium. ID, gene identifier; log_2_FC, mean of unigenes with the same PFAM; Total, number of unigenes from the same PFAM; PFAM, name of the protein family to which the enzyme it encodes belongs (obtained from the description of the eggNOG-mapper website).

	Regul	PFAM	log_2_FC	*p*-_value_	Total	Description
*A. acris*	Up	Chitin_bind_4	6.69	0.0056	4	Structural constituent of cuticle
		Protease serine-type, Rhomboid	3.07	0.0091	4	Serine-type endopeptidase activity. It is involved in the biological process described with proteolysis
		ADAM_spacer1,Pep_M12B_propep, Reprolysin,TSP_1	7.85	0.0015	1	Involved in the biological process related to proteolysis
		Hemocyanin_C, Hemocyanin_M, Hemocyanin_N	3.98	0.0016	2	Hemocyanin, all-alpha domain
		Cytochrom_B_C, Cytochrome_B	1.73	0.0262	1	Component of the ubiquinol-cytochrome c reductase complex that is part of the mitochondrial respiratory chain. The b-c1 complex mediates electron transfer from ubiquinol to cytochrome c
		P450	8.42	0.0091	1	Heme binding. It is involved in the biological process described with oxidation-reduction process
		COX3	2.57	0.0494	1	Subunits I, II and III form the functional core of the enzyme complex. Component of the cytochrome c oxidase, the last enzyme in the mitochondrial electron transport chain, drives oxidative phosphorylation
	Down	SNARE	−6.61	0.0421	1	SNARE domain: Facilitate the fusion of vesicles, responsible for the transport of molecules
*A. ludens*	Up	Chitin_bind_4	7.83	0.0097	37	Structural constituent of cuticle
		Trypsin, CLIP, Peptidase_S28, Peptidase_S10	4.75	0.0039	21	Serine-type endopeptidase or carboxypeptidase activity. It is involved in the biological process described with proteolysis
		Hemocyanin_C, Hemocyanin_M, Hemocyanin_N	3.45	0.0258	10	Oxidoreductase activity. It is involved in the biological process described with metabolic process. Hemocyanin, all-alpha domain
		3HCDH, 3HCDH_N	3.37	0.0018	5	3-hydroxyacyl-CoA dehydrogenase activity. It is involved in the biological process described with oxidation-reduction process
		6PGD, NAD_binding_2	3.21	0.0004	1	Catalyzes the oxidative decarboxylation of 6- phosphogluconate to ribulose 5-phosphate and CO(2), with concomitant reduction of NADP to NADPH
		adh_short_C2	4.66	0.0003	4	Oxidoreductase activity. It is involved in the biological process described with metabolic process
		Aldo_ket_red	2.43	0.0388	4	It is involved in the biological process described with oxidation-reduction processes
		p450	3.74	0.0001	3	Heme binding. It is involved in the biological process described with oxidation-reduction process
		Cuticle protein	4.81	0.0027	3	Cuticle protein
		GST_C, GST_C_3, GST_N, GST_N_3	1.92	0.0130	2	Conjugation of reduced glutathione to a wide number of exogenous and endogenous hydrophobic electrophiles
		Malic_M, malic	1.71	0.0051	1	It is involved in the biological process described with oxidation-reduction processes
		G6PD_C, G6PD_N	2.12	0.0496	1	Catalyzes the rate-limiting step of the oxidative pentose-phosphate pathway, which represents a route for the dissimilation of carbohydrates besides glycolysis
	Down	PI3K_C2,PI3K_p85B,PI3K_rbd,PI3Ka,PI3_PI4_kinase	−6.93	0.0056	2	Phosphotransferase activity, alcohol group as acceptor. It is involved in the biological process described with phosphatidylinositol phosphorylation
		FAT,FATC, PI3_PI4_kinase	−7.27	0.0027	1	Belongs to the PI3 PI4-kinase family
		C2, HECT,WW	−6.5	0.034	1	Ubiquitin-protein transferase activity

**FIGURE 4:**
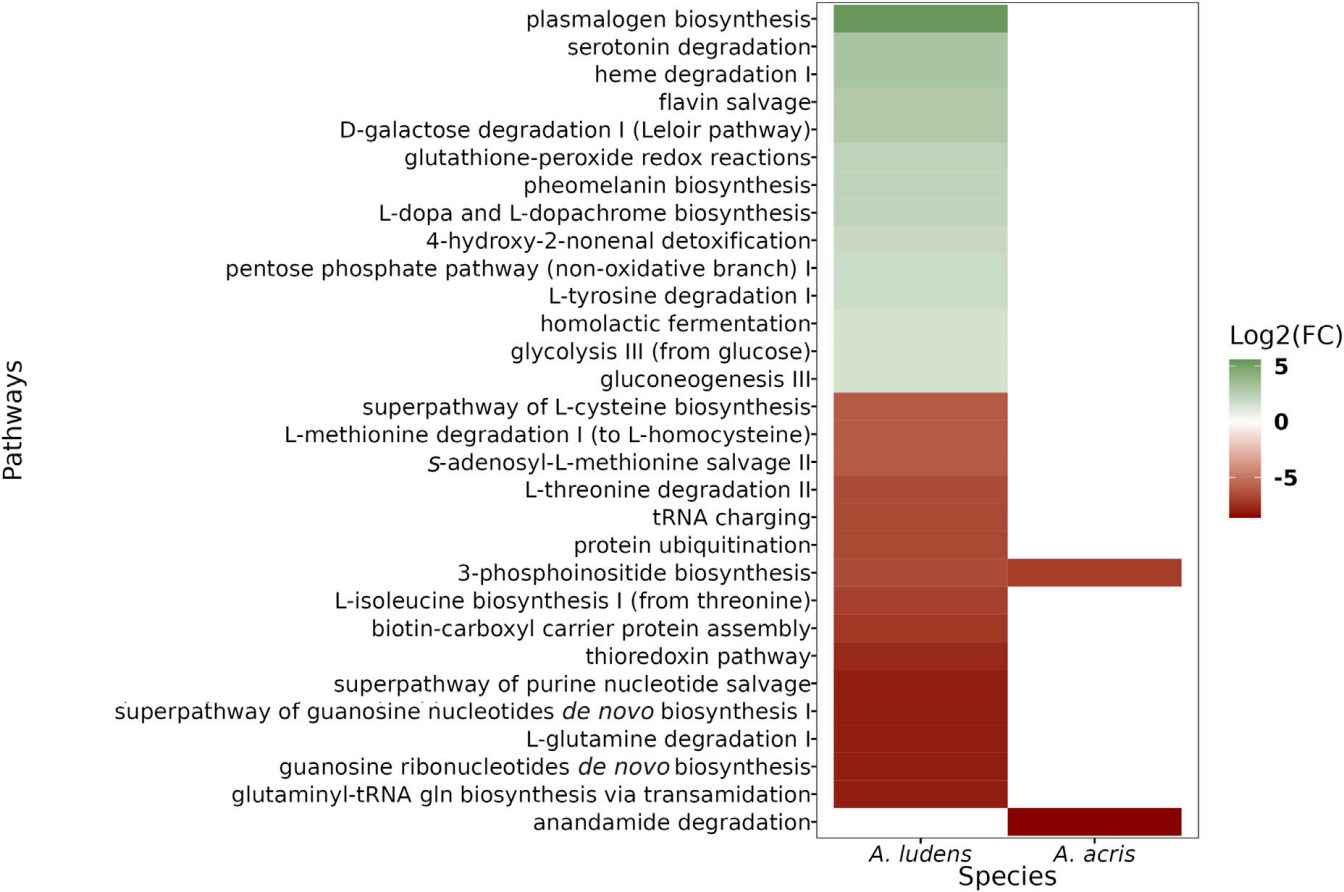
Heatmap of differential metabolic pathways found in *A. acris* and *A. ludens* larvae that were reared in an artificial diet containing 6% *H. mancinella* pulp lyophylizates. The activated pathways are shown in green (or shades thereof) and the repressed ones, in ochre red.

We found a larger number of differential pathways mapped in *A. ludens* compared to *A. acris* (Figure 4). In *A. ludens*, we observed the activation of detoxification (*i.e*., Glutathione-peroxide redox reactions, 4-hydroxy-2-nonenal detoxification), pigment (*i.e.,* Pheomelanin biosynthesis, and L-dopa and L-dopachrome biosynthesis), and fermentative metabolic pathways (*i.e.*, glycolysis III, gluconeogenesis III, homolactic fermentation, D-galactose degradation I, and the pentose-phosphate pathway). Also, in *A. ludens* we observed the repression of the protein ubiquitination process, transcripts encoding for proteins involved in the biosynthesis and degradation of amino acids (including Methionine, Isoleucine, Cysteine, Threonine, and Glutamine) and the 3-phosphoinositide biosynthesis (PI3-K); this last (PI3K) was also repressed in *A. acris* (Figure 4). The only route repressed exclusively in *A. acris* was the anandamine degradation pathways (Figure 4).

## 4 Discussion

The study of the molecular responses of herbivorous insects to plant chemical defenses can be useful in our quest to better understand the mechanisms/metabolic routes that are activated to cope with toxic allelochemicals. Using a transcriptomic approach, here we sought to identify the regulated genes in the larvae of two closely phylogenetically related fruit flies with contrasting feeding habits (*i.e.*, monophagous vs. polyphagous), *A. acris* and *A. ludens*, when they are exposed to the pulp of *H. mancinella*, a plant that produces fruit that is highly toxic. In both species, an alteration was observed in the levels of transcript expression coding for enzymes related to cuticle biosynthesis, hypoxia, protease activity (trypsin and serine proteases) and metabolism of xenobiotics (*i.e.*, responses to abiotic stress) (Table 2). However, a higher number of these transcripts were only recorded in *A. ludens* compared to *A. acris*, the species that thrives in *H. mancinella* (Table 2). In addition to these transcripts found, we were able to map the phosphoinositol biosynthesis pathway (PI3K) in both species (Figure 4). On the other hand, activation in routes related to fermentative metabolism (energy production) and repression of pathways involved in the process of ubiquitination and biosynthesis and degradation of amino acids was only identified in *A. ludens* (Figure 4). The only pathway exclusively affected in *A. acris* was the degradation of anandamides (Figure 4). As will be discussed in what follows, it is likely that the lower regulation observed in *A. acris*, the strict specialist, is likely due to the fact that: 1) larvae of this species have the resistance mechanisms to effectively counteract the negative effects of the toxic allelochemicals present in *H. mancinella* fruit; 2) the low concentration of *H. mancinella* lyophilizate mixed in the diet in which the larvae were reared did not stress the larvae of *A. acris* enough as in nature they are exposed to higher concentrations (an aspect we are currently investigating); 3) these larvae might possibly have a symbiotic intestinal microbiota that facilitates the metabolization of the toxic compounds present in the pulp of *H. mancinella* (something we will research in a future study). Contrary to our prediction, it appears that *A. ludens*, the sister species that evolved into a broad generalist, has maintained most of the broad resistance mechanisms that it shares with *A. acris* which were expressed when exposed to the toxic diet it does not encounter in nature. But *A. ludens* appears to not have developed the specialized biochemical pathways or structural defensive mechanisms *A. acris* possesses that enable it to thrive in the highly toxic *H. mancinella*. A comparative genomics study would be necessary to shed definitive light on this.

The fruit of *H. mancinella* is rich in bioactive polyphenols, such as the ellagitannin Hippomannin A, considered the most toxic compound present in this fruit (Schaeffer et al., 1954; Lauter and Foote, 1955; Rao, 1974; Rao, 1977; Barbehenn et al., 2006; Aluja et al., 2020). The first defense line of insects to face these secondary metabolites is the cuticle. The larval cuticle is a physical barrier that can limit the passage of toxic compounds from the environment to the hemolymph (Pryor et al., 1947; Barrett, 1983; Ashida and Brey, 1995; 1997). We found upregulation of transcripts that encode for cuticle proteins (chitin-binding proteins) in both species (Table 2) and could be related to a response leading to the reinforcement of the cuticle of the larvae. In insects, an increase in the thickness of the cuticle has been related to resistance against secondary metabolites present in plants and insecticides (Balabanidou et al., 2018). For example, when larvae of *Spodoptera frugiperda* were exposed to camptothecin (a pentacyclic quinoline alkaloid isolated from *Camptotheca acuminata* Decne.), histopathological changes were observed in the midgut structure, followed by upregulation of several genes encoding for cuticular proteins (Shu et al., 2021). Consistent with this, we found that of the upregulated cuticle-proteins, 28 were not orthologous in *A. ludens*, and four were not orthologous in *A. acris*. We note, however, that many of these Unigenes were partial, and this could have biased the resolution of the orthology detection procedure followed. If the above is true, then the damage generated by the pulp of lyophilized *H. mancinella* could first occur through cuticular disruption. Considering this, *A. acris* can likely reinforce the resistance against *H. mancinella* by adapting its cuticle through structural proteins in the cuticle that are more efficient than those found in *A. ludens*, a species whose larvae are not naturally exposed to the toxicants in the fruit of this plant.

We also identified many transcripts encoding proteases, including trypsin (Table 2). Trypsin is a non-specific serine endopeptidase responsible for the maturation of various zymogens such as prophenoloxidase (important for immune responses) (Ashida and Brey, 1997; Perdomo-Morales et al., 2007; Soares et al., 2011; Lu et al., 2014; Wu et al., 2015). Prophenoloxidase is synthesized in insect hemocytes, actively transported into the cuticle through the epidermis, and randomly distributed in the endocuticle of the body wall (Ashida and Brey, 1997). Since phenoloxidase (mature version of phenoloxidase) is central to the immune response, as it can oxidize phenolic compounds to induce melanization of the cuticle, it is likely that the phenoloxidase system actively participates as a defense mechanism in response to *H. mancinella* compounds (Lu et al., 2014; Wu et al., 2015).

In insects there is an antagonistic interaction between the ingestion of phenols and the assimilation of nutrients, which can cause an inhibition of the digestion of carbohydrates or proteins, affecting their nutrition, growth, and development (Schopf, 1986; Felton et al., 1992; Haukioja et al., 2002; Pascacio-Villafán et al., 2016; Punia et al., 2020; Thitz et al., 2020). This antagonistic interaction is produced due to the inhibition of digestive enzymes by polyphenols. In the case of ellagitannins, it has been documented that they can inhibit alfa-amylase and trypsin activity (McDougall and Stewart, 2005). Many phytophagous insects counteract the polyphenols' effect by a high protease synthesis rate (Brioschi et al., 2007; Spit et al., 2014; Zhu-Salzman and Zeng, 2015), a mechanism we identified in both fly species in this study. We identified the upregulation of trypsin and serine proteases, 21 in *A. ludens* and four in *A. acris*. However, our results suggest differences in these enzymes in both flies, with two peptidases in *A. acris* not being orthologous with *A. ludens*. On the other hand, 13 peptidases present in *A. ludens* were not orthologous in *A. acris*.

Interestingly, one non-orthologous serine-type endopeptidase in *A. acris* is a rhomboid-like1 protein. Rhomboid serine proteases, together with ADAM (a disintegrin and metalloproteinase) proteins, are involved in the Epidermal Growth Factor Receptor (EGFR) pathway, catalyzing the cleavage of membrane-tethered epidermal growth factor (EGF)-like ligands, being a regulator and activator of EGFR (Lee et al., 2001; Urban, 2002; Blobel, 2005). In *A. acris*, a homologous of the ADAM metalloprotease was upregulated (Unigen: acris005570). In *Drosophila*, EGFR regulates the penetration resistance to the insecticide avermectin, regulating the chitin synthesis and promoting the increment of peritrophic membrane thickness in larvae resistant to avermectin (Chen et al., 2016). Interestingly in human cells, EGFR activation can be inhibited by epigallocatechin-gallate, triggering the internalization of the EGFR (Adachi et al., 2007; Adachi et al., 2008). Even though the EGFR pathway seems conserved in all eukaryotes, differences could exist between *A. ludens* and *A. acris*, based on the difference in the rhomboid protein, that hypothetically could be related to the maturation of the EGFR-ligand.

When *A. ludens* larvae are reared in diets or fruit containing high concentrations of phenolic compounds (*e.g.*, *Malus domestica* Borkh), a reduction in egg production, larval weight, and an increase in larval development and pupation times has been recorded (Aluja et al., 2014; Pascacio-Villafán et al., 2014). The induction of deformities was also observed in pupae, as well as an increase in mortality and the duration of the biological cycle, which lasts up to three times more compared to larvae reared on natural hosts such as grapefruit, with a lower content of phenolic compounds (Aluja et al., 2014; Pascacio-Villafán et al., 2014). At a molecular level, phenolic compounds bind to the amino acid-building proteins in the diet and/or to digestive tract enzymes (Schopf, 1986; Felton and Duffey, 1991). This happens because the aromatic rings of amino acids and polyphenols interact through π-type interactions, destabilizing the tridimensional structure of proteins, which induces protein denaturation and coalescence with other proteins (Adamczyk et al., 2012; Chen et al., 2019). These effects inactivate the function of the proteins affected, and the biological processes in which they participate (Haslam, 2007; Spalinger et al., 2010; Adamczyk et al., 2012). Tannins, a particular class of polyphenols, can produce high levels of semiquinones and quinones, which affect the redox status of the cell contributing to poor absorption of nutrients and generation of cytotoxic effects in the affected tissues (Appel, 1993; Salminen et al., 2004; Barbehenn et al., 2005; Barbehenn and Constabel, 2011).

In addition, and in contrast to what was observed in *A. acris*, in *A. ludens* a high number of transcripts that encode for proteins involved in energy processes and in the biosynthesis and degradation of amino acids were found (Figure 4). So, not surprisingly, the larvae of *A. ludens* suffered more metabolic changes than *A. acris*. For example, we observed the activation of fermentative pathways, inferring that energy metabolism was one of the most affected aspects in *A. ludens*. This effect was not observed in *A. acris*, suggesting that the basal defense mechanisms of *A. acris* were sufficient to prevent the metabolic effects observed in *A. ludens*.

The transcriptional responses of both phylogenetically related species involve the activation of transcripts that encode for similar enzymes, such as enzymes with heme-binding sites, proteases, or chitin-binding enzymes (Table 2). It is likely that the high concentration of tannins present in the pulp of *H. mancinella*, together with the metal chelating activity of the o-dihydroxyphenyl groups of tannins, causes a decrease in the bioavailability of iron, copper, and other metallic macro or micronutrients (McDonald et al., 1996; Afsana et al., 2004; Fiesel et al., 2015; Delimont et al., 2017). So, if tannins were chelating metals, proteins that have an active site coordinated with metal ions, such as those with heme groups, would become dysfunctional. For example, the activation of genes that encode for hemocyanin (a heme-containing protein) could cause a decrease in the oxygen transport activity through the hemolymph (Engel and Brouwer, 1991; Martin and Hose, 1995). Therefore, if the tannins are interfering with the oxygen transport activity, and favoring hypoxic conditions, a plausible countermeasure to compensate hypoxia could be the transcriptional activation of hemocyanins (Engel and Brouwer, 1991; Martin and Hose, 1995; Salminen and Karonen, 2011). Also, the decrease in the concentration of oxygen available in the hemolymph would explain the activation of the fermentative pathways observed in *A. ludens*. Usually, the activation of fermentative pathways leads to the rapid production of energy, but this process is insufficient when these conditions are maintained for a long time, affecting the metabolic performance of the organism (Hoback and Stanley, 2001).

In both species, the activation of genes coding for proteins related to the metabolism of xenobiotics and in oxidation-reduction processes was also found (Table 2). As was the case with other defensive responses, in *A. ludens* the number of these transcripts was much higher compared to *A. acris*. In this sense, the activation of transcripts related to the first stage of xenobiotic metabolism is relevant, since these enzymes represent one of the first lines of defense against toxic compounds (Li et al., 2007). So, the activation of transcripts encoding for P450 monooxygenase enzymes, glutathione-S-transferases, and transporters could be related to the coordinated response of detoxification pathways and toxin excretion (Chahine and O'Donnell, 2012; Zheng et al., 2022). In future studies, it would be of interest to identify the anatomical site where these transcripts are altered.

Repression of signaling via phosphoinositides was also identified in both species. For example, in *A. ludens* the transcript of phosphatidylinositol 4, 5-bisphosphate kinase (PI3K) was specifically repressed (Figure 4). PI3K is an important regulator of cell growth in insects, as it inhibits the signaling that leads to a reduction in body size, tissue growth, and starvation (Britton et al., 2002; Lin and Smagghe, 2019). This happens because PI3K activity is regulated by the availability of protein in the diet and coordinates cellular metabolism based on the nutritional conditions of the individual (Britton et al., 2002).

Notably, in *A. acris*, the anandamide degradation pathway was repressed (Figure 4). Anandamide is a bioactive fatty acid amide widely studied in mammals that functions as a messenger molecule regulating feeding behavior (*e.g.*, hunger, and satiety perception), among other functions (Sharkey et al., 2014). However, in insects, there are few studies on the metabolism of anandamide and its functions (Ban et al., 2003; Farrell and Merkler, 2008; Tortoriello et al., 2013; Jeffries et al., 2014).

In other study models such as the plant *Arabidopsis thaliana*, it has been reported that fungi toxin elimination occurs via a vesicle-mediated transport which is translocated from the plasma membrane to vacuoles (Wang et al., 2020). This mechanism, which is not exclusive to plants, could occur also in insects in which the detoxification strategy includes catabolism and the sequestration of plant-defense secondary metabolites. The above can explain, at least in part, why proteins like SNARE are differentially expressed. SNARE proteins represent a superfamily of small, mostly membrane-anchored proteins that catalyze the fusion of membranes in all eukaryotic cells (Margiotta, 2022).

## 5 Conclusion

The larvae of *A. acris* and *A. ludens* responded at a transcriptional level to counteract the negative effects caused by the ingestion of *H. mancinella* pulp, expressing overall similar mechanisms. However, in *A. ludens* the number of transcripts altered was much greater than in *A. acris*. These transcriptional responses of both species were mainly related to hypoxia, cuticle biosynthesis, and xenobiotic metabolism. Our results indicate that *A. acris* can easily handle a low level of toxicity in *H. mancinella* lyophilizates added to an artificial diet (6%), but additional experiments with higher concentrations of the toxicants in the fruit are needed to fully understand the biochemical/molecular mechanisms larvae of this species use to degrade or block these deleterious allelochemicals. From our results it becomes clear that during the divergence of these phylogenetically close species, *A. acris* developed adaptations to deal with the toxicants of its only known host, whereas *A. ludens*, following another evolutionary path, maintained certain common biochemical routes, expressed here when exposed to the toxicants of a fruit that is not a host in nature, and apparently lost others that inhibit its development in *H. mancinella*. But further research is needed to clarify if *A. acris* indeed evolved novel toxicant degradation/blocking routes, or if its gut contains symbiotic bacteria that are the ones that metabolize the toxicants. This is needed as in this study we studied the transcriptome of entire larvae, including the gut. In future studies we will concentrate on the gut and the bacteria contained in it. Further research is also needed on possible morphological adaptations in the midgut of the larvae (e.g., thickness of gut walls) through which the entrance of toxicants via the gut into the hemolymph could be blocked.

## Data Availability

The raw datasets for this study can be found in the Sequence Read Archive (SRA) of the National Center for Biotechnology Information (NCBI) under the accession number PRJNA957832.
