# Supplementary figures and images for: Insights into the differences related to the resistance mechanisms to the highly toxic fruit Hippomane mancinella (Malpighiales: Euphorbiaceae) between the larvae of the sister species Anastrepha acris and Anastrepha ludens (Diptera: Tephritidae) through comparative transcriptomics
Source: Front Physiol. 2024 Jan 18;15:1263475. doi: 10.3389/fphys.2024.1263475 (PMC10830740; doi:10.3389/fphys.2024.1263475)

## Supplementary Material 2

### Graphical abstract

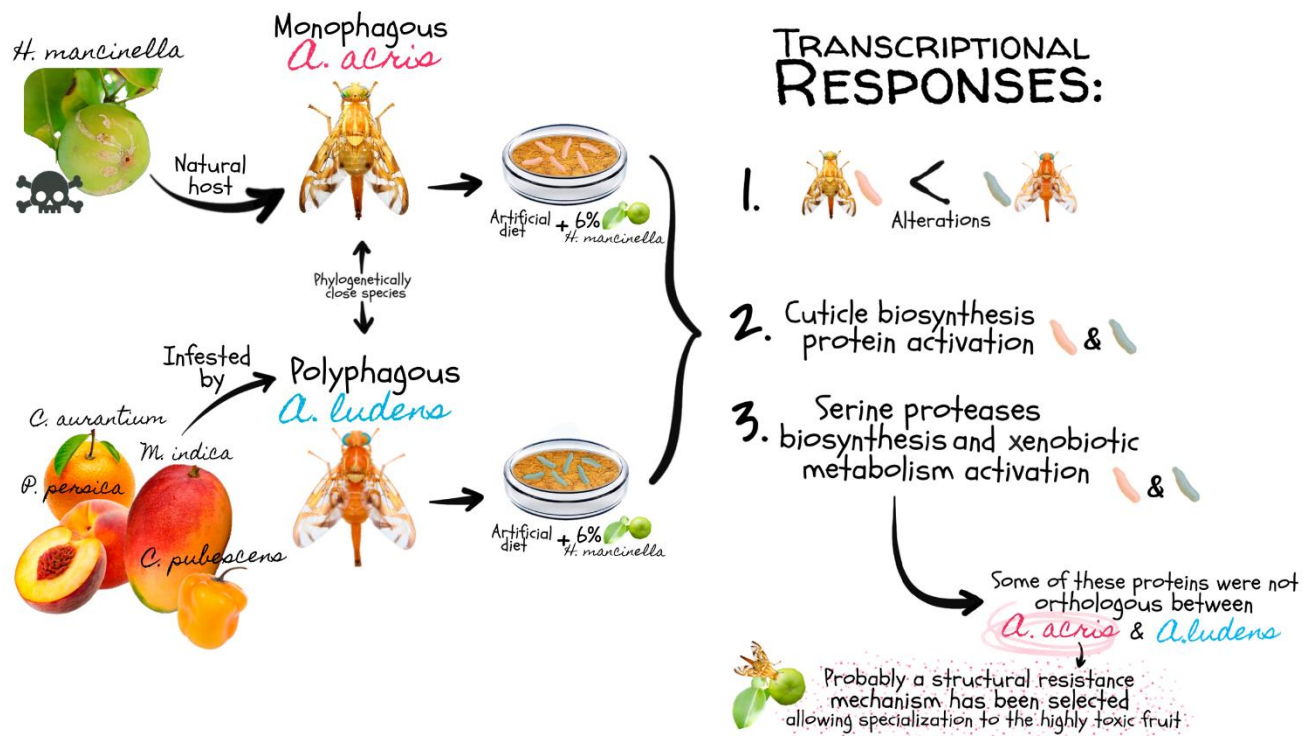

Supplement: Supplementary file 1 [file Image2.pdf]
