# Supplementary material for: Insights into the differences related to the resistance mechanisms to the highly toxic fruit Hippomane mancinella (Malpighiales: Euphorbiaceae) between the larvae of the sister species Anastrepha acris and Anastrepha ludens (Diptera: Tephritidae) through comparative transcriptomics
Source: Front Physiol. 2024 Jan 18;15:1263475. doi: 10.3389/fphys.2024.1263475 (PMC10830740; doi:10.3389/fphys.2024.1263475)
Supplement: Supplementary file 2 [file Image1.pdf]

# *Supplementary Material*

## RNA Quality of the RNA extractions of both experiments

RNA with sufficient concentration and integrity were obtained for all samples (Supplementary Fig. 1).

## Supplementary Figure

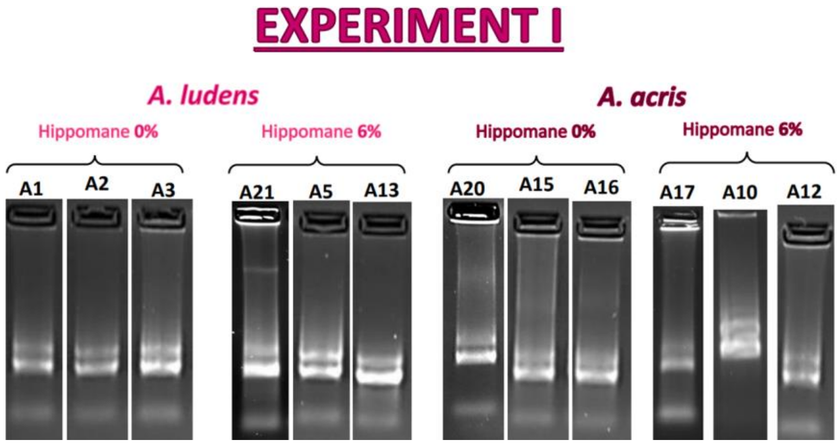


**Supplementary Figure 1.** Agarose (1%) gel electrophoresis of total RNA extractions of the larval samples exposed to the different treatments. Bands were visualized with UV light on a BioRad ChemiDoc MP imager.
